# 421. Acute Rheumatic Fever at a US Children's Hospital During a Period of Fluctuating Group A *Streptococcus* Incidence

**DOI:** 10.1093/ofid/ofae631.135

**Published:** 2025-01-29

**Authors:** Jonathon C McNeil, Richard Bui, Tam Doan, Marietta DeGuzman, Misu A Sanson, Anthony R Flores, Lauren M Sommer

**Affiliations:** Baylor College of Medicine, Houston, TX; Baylor College of Medicine, Houston, TX; Baylor College of Medicine, Houston, TX; Baylor COM, Houston, Texas; McGovern Medical School, Houston, TX; Vanderbilt University Medical Center, Nashville, TN; Baylor College of Medicine, Houston, TX

## Abstract

**Background:**

Several centers have reported shifts in the incidence of invasive Group A *Streptococcus* (GAS) in children with a decline in the early SARS-CoV 2 pandemic followed by a surge in disease in 2022-2023. Acute rheumatic fever (ARF) is a well-known complication of GAS pharyngitis with low incidence in North America. We reviewed cases of ARF at a tertiary US children’s hospital during a period of changing GAS activity.

**Figure:**
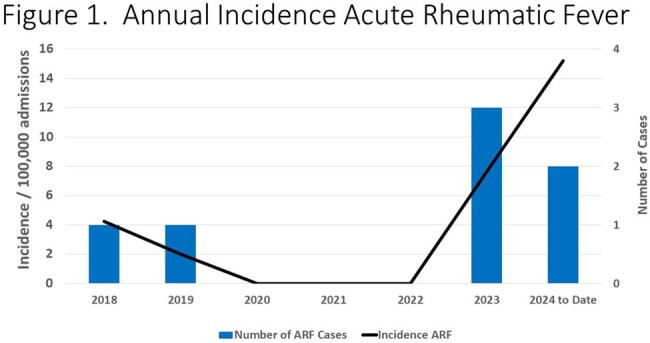

**Methods:**

Cases of ARF from Jan 2018-Mar 2024 at Texas Children’s Hospital (TCH) were identified using ICD10 codes. Cases with initial diagnosis of ARF at an outside institution were excluded. Hospital admissions data were used to estimate ARF incidence. We also reviewed all culture positive infections captured by an active GAS surveillance study at TCH. This was supplemented by enumeration of positive GAS antigen tests across the TCH system (3 hospitals, 12 urgent cares and 51 primary care offices). Temporal trends in GAS disease were examined.

Quarterly Trends in ARF and Throat Streptococcal Antigen Positivity

**Figure:**
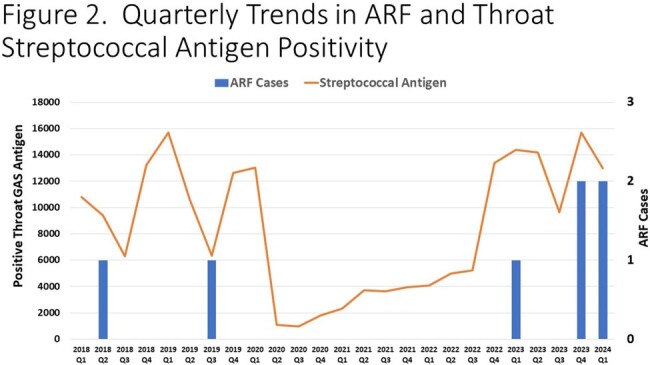

Positive streptococcal antigen testing represents that across the entire Texas Children's Hospital system including inpatient, outpatient, emergency room and urgent care encounters.

**Results:**

1,839 culture positive GAS infections and 803 culture positive GAS pharyngitis were identified. 210,104 throat streptococcal antigen tests were positive in the study period. Significant shifts occurred in GAS culture and rapid antigen test positivity during the study period with a decline in 2020-2021 (p=0.003 and p=0.006, respectively), followed by an increase in cases in the subsequent years (p=0.01 and p=0.03). During the study period, seven cases of ARF were identified. No cases occurred during the interval from 2020-2022 followed by five cases (71.4%) in 2023-Q1 2024 (**Figure 1**), mirroring overall trends in GAS (**Figures 2-3**). The median age of ARF cases was 8.3 years. All cases were associated with carditis (**Figure 4**), mostly commonly involving the mitral valve. All cases in 2023-2024 had severe, symptomatic mitral regurgitation. Two subjects required cardiac surgery. Three subjects had chorea.

**Figure:**
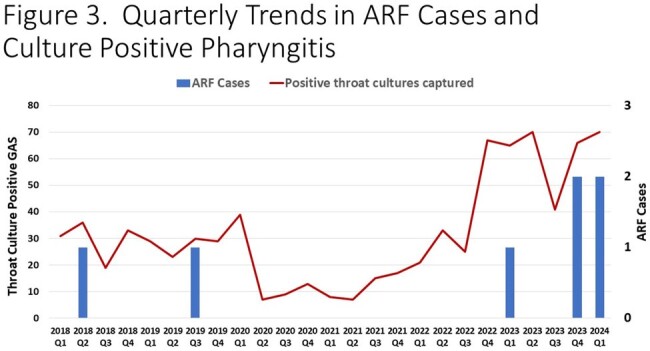

Culture positive pharyngitis cases are those captured by the GAS surveillance study at Texas Children's Hospital. All culture positive pharyngitis cases were reviewed to confirm diagnosis.

**Conclusion:**

While ARF remains an uncommon diagnosis in North American populations, we observed a cluster of severe ARF in late 2023-early 2024 coinciding with an increase in GAS pharyngitis. Although the overall risk remains small, clinicians should remain vigilant for this complication particularly during the global increase in GAS activity. Additional work utilizing multicenter data sets are needed to further assess these findings.

**Figure:**
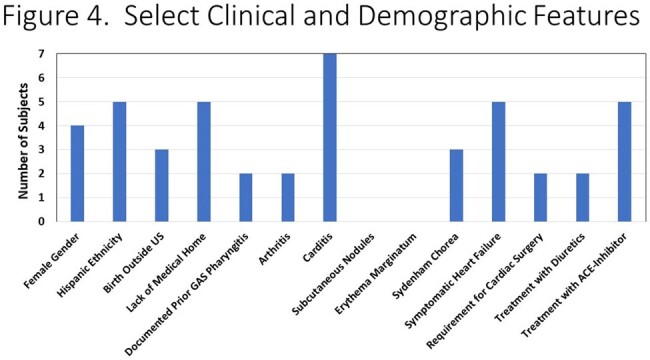

**Disclosures:**

**Jonathon C. McNeil, MD**, Nabriva: site investigator on clinical trial **Anthony R. Flores, MD, MPH, PhD**, GlycosBio, Inc: Grant/Research Support

